# Parietal Alpha-ERD and Theta-ERS Serve as Neuroelectrical Indices for Working Memory Impairment Following Total Sleep Deprivation

**DOI:** 10.3390/brainsci16030333

**Published:** 2026-03-20

**Authors:** Wenbin Sheng, Zihan Gang, Liwei Zhang, Yongcong Shao, Qianxiang Zhou

**Affiliations:** 1 Key Laboratory of Biomechanics and Mechanobiology (Beihang University), Ministry of Education, Key Laboratory of Innovation and Transformation of Advanced Medical Devices, Ministry of Industry and Information Technology, National Medical Innovation Platform for Industry-Education Integration in Advanced Medical Devices (Interdiscipline of Medicine and Engineering), School of Biological Science and Medical Engineering, Beihang University, Beijing 100191, China; bf2310243@buaa.edu.cn; 2 School of Psychology, Beijing Sport University, No 48, Xinxi Road, Haidian District, Beijing 100084, China; 2024210211@bsu.edu.cn (Z.G.); budeshao@bsu.edu.cn (Y.S.); 3 Chinergy Co., Ltd., Beijing 100193, China; karenzlw1@163.com

**Keywords:** sleep deprivation, working memory, alpha-ERD, theta-ERS

## Abstract

**Highlights:**

**What are the main findings?**

- A 36 h duration of TSD significantly impairs verbal working memory accuracy and reaction time.
- TSD induces abnormal enhancements of parietal alpha-ERD/theta-ERS and suppressed N2/P3 amplitudes.

**What are the implications of the main findings?**

- Enhanced parietal ERD/ERS reflects an inefficient neural compensatory mechanism under fatigue.
- Parietal electrophysiological indices serve as reliable biomarkers for sleep loss-induced working memory impairment.

**Abstract:**

**Background/Objectives:** Acute total sleep deprivation (TSD) is known to impair working memory capacity. However, the specific relationship between alterations in the brain’s electrical power spectrum following TSD and working memory deficits remains poorly understood. **Methods:** In this study, 30 healthy young adults (14 males and 16 females) were enrolled, and 28 participants were finally included in the analysis after excluding EEG data with excessive noise, who underwent a verbal working memory task under two conditions: baseline sleep (BL) and 36 h of TSD. EEG data were recorded concurrently. **Results:** We observed a significant decrease in working memory accuracy and a significant prolongation of reaction time after TSD. Furthermore, TSD led to a significant enhancement of parietal alpha-ERD (at electrodes P3/Pz/P4) and theta-ERS, accompanied by a reduction in N2 and P3 wave amplitudes. **Conclusions:** These findings suggest that TSD may impair working memory by weakening parietal alpha-ERD and early conflict monitoring and late attention evaluation processes. The enhanced theta-ERS might represent a compensatory mechanism.

## 1. Introduction

Sleep deprivation (SD) represents a substantial global public health concern. Increased demands from overtime work or specific professional obligations frequently compel individuals to adopt lifestyles characterized by late bedtimes and early awakenings, thereby diminishing their essential sleep duration. This pervasive phenomenon exerts direct detrimental effects on both physical and mental health, contributing to emotional dysregulation, obesity, and augmented risks of hypertension and cardiovascular disease [1,2,3,4]. Consequently, research investigating sleep deprivation has become a focal area of contemporary scientific inquiry.

Among the diverse adverse consequences of SD on physiological and psychological well-being, its detrimental impact on cognitive function is particularly salient. Both acute sleep deprivation (defined as 24 consecutive hours without sleep) and chronic sleep insufficiency (characterized by long-term daily sleep of less than 6 h) demonstrably impair cognitive abilities such as alertness, attention, memory, and decision-making efficiency [5,6]. Working memory (WM), a cognitive system characterized by limited capacity for information storage and manipulation, underpins higher-order cognitive processes including comprehension, learning, and reasoning [7]. WM is integral to maintaining and manipulating information [8] and functions as a critical intermediary between short-term and long-term memory systems [9]. Significantly, WM is identified as one of the earliest cognitive domains affected by SD [10] and experiences the most profound impairment among various cognitive functions [11].

Studies indicate that both acute sleep deprivation and chronic sleep insufficiency can significantly inhibit hippocampal neuronal activity, thereby impairing the transition from short-term to long-term memory [12]. Furthermore, drift-diffusion models reveal that sleep deprivation affects nearly all information processing stages in working memory (WM) tasks [13]. An fMRI study found that the decline in WM performance post-sleep deprivation (SD) was negatively correlated with enhanced functional connectivity (FC) between specific brain regions, including the left cerebellar culmen and right inferior parietal lobule, left cuneus and left lingual gyrus, and left cuneus and right posterior cingulate gyrus [14]. Despite these findings, the specific neural mechanisms underlying SD’s impact on WM remain poorly understood, particularly concerning event-related electrophysiological activities. While fMRI has yielded interesting results, its high spatial but low temporal resolution limits its ability to capture the rapid, dynamic neural changes during WM tasks. Consequently, neuroimaging techniques with high temporal resolution are needed to directly reflect dynamic, task-related neural activity and further elucidate how sleep deprivation affects working memory.

Event-related desynchronization (ERD) and event-related synchronization (ERS) are advanced techniques for analyzing brain function changes and offer unique insights in this field [15]. These phenomena are closely related to event-related spectral perturbation (ERSP), which quantifies the relative change in the EEG spectrum (compared to spontaneous EEG) over time–frequency domains [16]. ERD/ERS represent the percentage change in power within task-specific frequency bands, identifiable from ERSP. Specifically, ERD/ERS manifest as positive spectral changes (event-induced EEG spectral components > spontaneous EEG) and negative spectral changes (event-induced EEG spectral components < spontaneous EEG). Different frequency bands of ERD/ERS are associated with distinct neural functions. Alpha-ERD is a well-established indicator of cortical neuronal population activation; a larger amplitude signifies greater neural activation and information processing engagement in that region [17]. Theta-ERS, on the other hand, is linked to cognitive processes like memory maintenance, information encoding, and attention [18,19].

Consequently, this study seeks to delineate the precise neural mechanisms by which sleep deprivation (SD) influences working memory (WM) and to ascertain the presence of any compensatory mechanisms. Our approach will involve analyzing alterations in WM task accuracy and reaction time following SD, alongside changes in parietal alpha-ERD amplitude (at electrode sites P3/Pz/P4) and other relevant electrophysiological markers (e.g., N2 and P3 waves). Building upon this, we posit the following hypotheses: (1) acute total sleep deprivation will result in a significant reduction in subjects’ working memory task accuracy and a significant prolongation of their reaction time; (2) acute total sleep deprivation will modify parietal ERD and ERS activities, with this effect being more pronounced under higher working memory task loads; and (3) sleep deprivation may attenuate the amplitude of the late positive component (P3), diminish early conflict monitoring resources (N2), reduce late update/evaluation resources (P3), and affect N2 latency, collectively indicative of compromised top-down attentional resource allocation efficiency.

## 2. Materials and Methods

### 2.1. Participants

A formal a priori power analysis was performed using G*Power 3.1 for the repeated measures ANOVA within-subject design. With effect size f = 0.25, α = 0.05, power = 0.80, 6 repeated measurements (2 conditions × 3 electrodes), and correlation among repeated measures = 0.5, the minimum required sample size was calculated as 19.

A total of 30 healthy adult participants (14 males, 16 females; mean age = 21.7 ± 1.9 years) were enrolled. During the 36 h total sleep deprivation (TSD) period, participants engaged in a working memory task while their electroencephalogram (EEG) data were recorded. EEG data from two participants exhibiting excessive noise were excluded, resulting in a final dataset from 28 participants for subsequent analysis. The final sample of 28 participants was sufficient to achieve adequate statistical power.

Participants were excluded from recruitment based on the following criteria: (1) non-right-handed; (2) abnormal visual acuity or corrected visual acuity; (3) age outside the range of 20–28 years; (4) Pittsburgh Sleep Quality Index (PSQI) ≥ 5 points evaluated before the experiment; (5) history of mental illness; (6) history of sleep-related diseases or major physical diseases; (7) use of sleep or psychotropic drugs within one month before the experiment; (8) habit of regularly drinking coffee, tea, or alcoholic beverages; (9) irregular work and rest schedules (daily sleep time outside the range of 7–9 h) within one week before the experiment.

This research protocol received approval from the Biomedical Ethics Committee of Beihang University (Ethical Code: BM20180040). All participants provided written informed consent prior to their participation and were remunerated monetarily following the successful completion of the study.

### 2.2. Experimental Procedure

**Figure 1:**
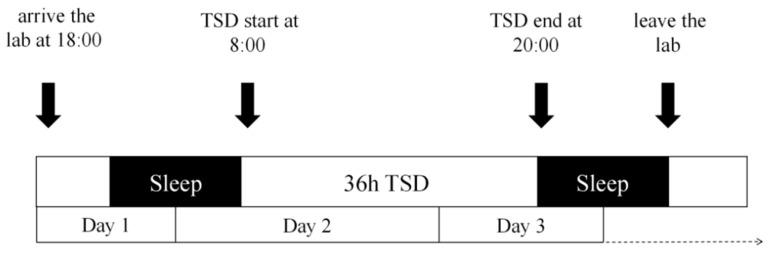
TSD experiment flow chart.

This study employed a within-subject design, where each participant completed two experimental conditions: baseline (BL) after normal sleep and 36 h of total sleep deprivation (TSD). A 3-week interval separated the conditions, with counterbalancing implemented. Figure 1 illustrates the TSD experimental flow. Participants arrived at the laboratory at 18:00 on Day 1 for an overnight stay. Following an explanation of the procedures and key considerations, they provided informed consent. The TSD period commenced at 08:00 on Day 2 and concluded at 20:00 on Day 3, with all data collection occurring at 20:00 on the final day.

During the study, participants adhered to strict guidelines: no drugs, alcohol, coffee, tea, milk tea, or other caffeinated beverages; avoidance of strenuous physical activity; and a regular sleep schedule. Sleep status was monitored through daily sleep logs, which confirmed that all participants retired before 22:00 nightly and achieved at least 7.5 h of sleep each night throughout the study.

### 2.3. Experimental Materials

**Figure 2:**
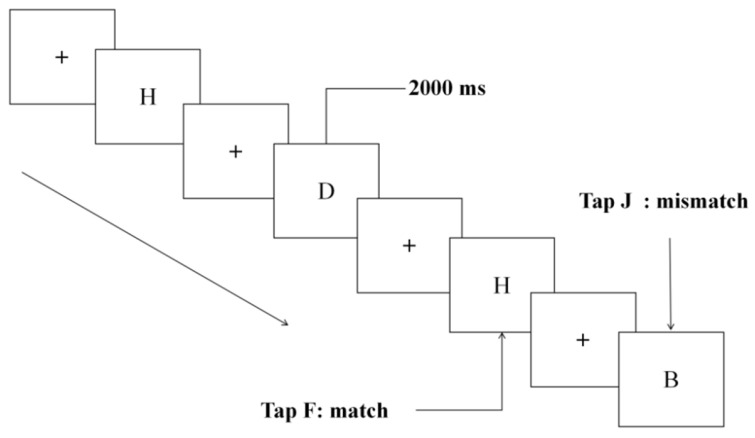
2-back experiment flow chart.

The experiment employed a 2-back task. Participants were instructed to determine if the currently presented stimulus congruented the stimulus presented two trials prior. In each trial, a fixation point “+” appeared centrally, followed by a black uppercase letter for 2000 ms, during which participants were required to respond. A trial was considered congruent if the presented letter matched the letter presented two trials prior; otherwise, it was incongruent (see Figure 2). The task comprised 120 trials, with an equal distribution of 60 congruent and 60 incongruent trials. Participants pressed the “F” key if the current letter was congruent with the letter two positions before, and the “J” key if it was not.

### 2.4. EEG Recording and Preprocessing

Electroencephalography (EEG) data were collected using a Scan 4.5 64-electrode system(Neuroscan, Inc., El Paso, TX, USA), adhering to the international 10–20 electrode placement standards. The sampling rate was set to 1000 Hz, with reference electrodes placed on both mastoids. Two horizontal electrooculography (EOG) electrodes were positioned two finger-widths below the eyebrows and approximately 1 cm from the outer canthi. Two vertical EOG electrodes were placed above the eyebrows and below the lower eyelids, respectively, to record eye movements. Skin impedance was reduced by exfoliating the skin with an abrasive paste prior to the application of bilateral mastoid and EOG electrodes.

EEG data preprocessing was conducted using Scan 4.3 software (Neuroscan, Inc., El Paso, TX, USA). Raw data were filtered with a 1–100 Hz band-pass filter and a 48–52 Hz notch filter to remove power line noise. Data were then segmented into 1500 ms epochs, time-locked from 500 ms before stimulus onset to 1000 ms after stimulus onset. Epochs were manually inspected: channels with significant signal drift were interpolated, and epochs containing obvious artifacts were discarded. Subsequently, eye movement artifacts were removed. Epochs with voltage values outside the range of −100 to 100 μV were excluded to ensure clean EEG data for further analysis.

### 2.5. Calculation of Event-Related Synchronization (ERS) and Event-Related Desynchronization (ERD)

This study estimated event-related synchronization/desynchronization (ERS/ERD) and event-related spectral perturbation (ERSP) by performing time–frequency decomposition (TFD) on multi-trial event-related EEG signals. The time–frequency distributions of individual trials were subsequently averaged to derive ERS/ERD values. The methodological workflow comprised the following steps: ① ensuring a sufficient number of trials by repeating experimental events of interest; ② preprocessing raw EEG data to remove artifacts and noise; ③ segmenting continuous EEG signals into individual trial epochs, time-locked to event trigger points; ④ performing time–frequency analysis on each trial to obtain its time–frequency representation; and ⑤ averaging the time–frequency representations across all trials and applying baseline correction to obtain the final ERS/ERD or ERSP estimates.

For TFD analysis on individual trials, custom MATLAB (MathWorks, Natick, MA, USA; v2018b) functions were utilized, implementing a sliding Fourier transform (WFT). A Hanning window of fixed length (250 ms) was applied, and the time–frequency plane spanned from −500 to 1000 ms (with a temporal resolution of 1 ms) and from 1 to 100 Hz (with a frequency resolution of 1 Hz). The resulting spectrogram, denoted as *P*(*t*,*f*) = |*F*(*t*,*f)*|^2^, quantifies the joint distribution of signal power in both time and frequency dimensions. To account for baseline activity, the spectrograms from all trials were averaged. The baseline reference interval was defined as −400 to −100 ms prior to stimulus onset, a period chosen to minimize boundary effects from the window function and interference from post-stimulus activity on spectral estimation. A subtraction method was employed for baseline correction to circumvent the positive bias potentially introduced by percentage-based normalization approaches.

### 2.6. Event-Related Potential (ERP) Analysis

To investigate the effect of sleep deprivation on working memory processing, event-related potentials (ERPs) were also calculated. Preprocessed EEG data were segmented into time windows from −200 ms to 800 ms post-stimulus onset, with baseline correction applied using the −200 ms to 0 ms interval. Valid trials under each condition were then time-locked and averaged to extract ERP waveforms. Based on previous research, the N2 (200–300 ms) and P3 (300–500 ms) components were selected as analysis indices, focusing on parietal electrode sites (P3, Pz, P4). Peak amplitudes and latencies of these components were statistically analyzed to evaluate the impact of sleep deprivation.

### 2.7. Statistical Analysis

Paired-sample *t*-tests were conducted in SPSS 27 to analyze reaction time and accuracy of the working memory task across sleep deprivation conditions.

For EEG data, repeated measures ANOVA was used to examine the effects of experimental conditions (congruent and incongruent trials, before and after sleep deprivation) on ERD/ERS amplitudes. Similarly, repeated measures ANOVA tested the effects of experimental conditions on N2 and P3 peak amplitudes and latencies. When Mauchly’s test of sphericity was violated, the Greenhouse–Geisser correction was applied. Bonferroni correction was used for significant interactions.

## 3. Results

### 3.1. Behavioral Analysis Results

Paired-samples *t*-tests were conducted to compare reaction time and accuracy on the working memory task before and after 36 h of sleep deprivation. Results indicated that sleep deprivation significantly decreased working memory accuracy and increased reaction time. Congruent condition: Significant differences were observed in both working memory accuracy (*M* = −0.548, *SD* = 0.130, *t*_(27)_ = 2.228, *p* = 0.034, 95% CI [−0.105, −0.004]) and reaction time (*M* = −87.687, *SD* = 204.877, *t*_(27)_ = 2.265, *p* = 0.032, 95% CI [−167.130, −8.244]) between pre- and post-deprivation. Incongruent condition: Significant differences were also found in working memory accuracy (*M* = −0.548, *SD* = 0.139, *t*_(27)_ = 2.083, *p =* 0.047, 95% *C*I [−0.109, −0.001]) and reaction time (*M* = −81.278, *SD* = 192.346, *t*_(27)_ = 2.236, *p* = 0.034, 95% CI [−155.862, −6.694]) between pre- and post-deprivation.

#### Exploratory Sex-Dependent Difference Analysis

To address potential sex dimorphism in sleep regulation and cognitive performance, we conducted a two-way ANOVA to explore sex-dependent differences in working memory behavioral indices and parietal electrophysiological indices under baseline and 36 h of TSD (after-sleep) conditions, across congruent and incongruent tasks. The cohort included 14 males and 14 females, with a significance level set at *p* < 0.05 for all analyses.

About behavioral results, no significant interaction of sex × TSD was observed in accuracy or RT (see Table 1). Simple effects analysis revealed no significant sex differences in all behavioral indices under BL or SD conditions (all *p* > 0.05). Regarding electrophysiological results, for parietal alpha-ERD and theta-ERS, no significant interaction of sex × TSD and no main effect of sex were observed across all electrode sites and task conditions (all *p* > 0.05; see Table 2).

Notably, the sex × TSD interaction for theta-ERS at Pz (congruent) and P4 (incongruent) approached marginal significance (Pz: *p* = 0.021; P4: *p* = 0.033), but post hoc tests confirmed no significant simple effects between sexes under pre-sleep or after-sleep conditions (all *p* > 0.05).

These results consistently indicate that 36 h of TSD impairs working memory and modulates parietal theta-ERS/alpha-ERD in a sex-independent manner in healthy young adults.

### 3.2. EEG Time–Frequency Analysis Results

**Figure 3:**
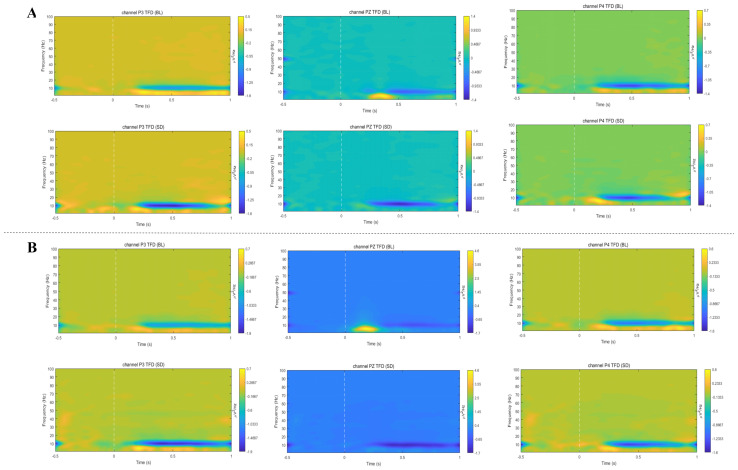
Time–frequency maps of ERD/ERS at parietal electrodes P3, Pz, and P4 under congruent (**A**) and Incongruent (**B**) conditions. Yellow colors represent event-related synchronization (ERS, increased spectral power relative to baseline), and blue colors represent event-related desynchronization (ERD, decreased spectral power relative to baseline). The horizontal axis represents time (ms, with 0 indicating stimulus onset), and the vertical axis represents frequency (Hz). The color bar on the right indicates the magnitude of ERD/ERS (in percentage change relative to baseline).

ERS/ERD results were obtained following the calculation method described in Section 2.5, as shown in Figure 3.

#### 3.2.1. ERS Results

Event-related synchronization (ERS) values were computed across the delta (1–4 Hz), theta (4–8 Hz), alpha (8–13 Hz), beta (13–30 Hz), and gamma (>30 Hz) frequency bands. Under the congruent condition, a significant main effect of time on theta-ERS was found (*F*(1, 27) = 8.29, *p* = 0.008, *η*^2^ = 0.24), demonstrating a significant enhancement of theta-ERS following 36 h of sleep deprivation. The main effect of electrode position on theta-ERS was not significant (*F*(1.37, 36.9) = 0.46, *p* = 0.57, *η*^2^ = 0.02), and the interaction between time and electrode position was also non-significant (*F*(1.23, 33.1) = 1.73, *p* = 0.199, *η*^2^ = 0.06).

In the incongruent condition, a significant main effect of time was also observed for theta-ERS (*F*(1, 27) = 6.46, *p* = 0.017, *η*^2^ = 0.19), with sleep deprivation leading to enhanced theta-ERS. The main effect of electrode position did not reach statistical significance (*F*(1.21, 33.9) = 0.11, *p* = 0.793, *η*^2^ = 0.004), nor did the interaction between time and electrode position (*F*(1.35, 37.7) = 1.60, *p* = 0.218, *η*^2^ = 0.05). Table 1 details the theta-ERS values obtained within the working memory task paradigm before and after sleep deprivation. No significant differences were detected in the remaining frequency bands.

#### 3.2.2. ERD Results

Event-related desynchronization (ERD) was computed across the delta (1–4 Hz), theta (4–8 Hz), alpha (8–13 Hz), beta (13–30 Hz), and gamma (>30 Hz) frequency bands. In the congruent condition, a significant main effect of time on alpha-ERD was identified (*F*(1, 27) = 5.55, *p* = 0.026, *η*^2^ = 0.17), with a notable enhancement of alpha-ERD following 36 h of sleep deprivation. Electrode position did not significantly influence alpha-ERD (*F*(1.66, 44.86) = 0.55, *p* = 0.535, *η*^2^ = 0.02), and the interaction between time and electrode position was also non-significant (*F*(1.55, 54) = 1.391, *p* = 0.257, *η*^2^ = 0.05).

For the incongruent task condition, time also demonstrated a significant main effect on alpha-ERD (*F*(1, 27) = 5.75, *p* = 0.024, *η*^2^ = 0.05), showing increased alpha-ERD after sleep deprivation. Electrode position did not yield a significant main effect (*F*(1.67, 45.1) = 0.87, *p* = 0.41, *η*^2^ = 0.03), nor did the interaction between time and electrode position (*F*(1.20, 32.44) = 3.05, *p* = 0.084, *η*^2^ = 0.10). Table 3 details the energy values of alpha-ERD during the working memory task before and after sleep deprivation. No significant differences were observed in the other frequency bands.

### 3.3. ERP Results

A 2 (sleep deprivation: BL, TSD) × 3 (electrodes: P3, Pz, P4) repeated measures ANOVA was performed on the peak amplitudes of the N2 (200–300 ms) and P3 (300–500 ms) components.

N2: In the congruent condition, a significant main effect of time was observed (*F*(1, 27) = 5.00, *p* = 0.034, *η*^2^ = 0.16), indicating a change in N2 amplitude after sleep deprivation. The main effect of electrode position (*F*(2, 54) = 0.21, *p* = 0.811, *η*^2^ = 0.02) and the time × electrode position interaction (*F*(2, 54) = 0.97, *p* = 0.394, *η*^2^ = 0.07) were not significant. Similarly, in the incongruent condition, time significantly affected N2 amplitude (*F*(1, 27) = 6.29, *p* = 0.018, *η*^2^ = 0.19). Neither the main effect of electrode position (*F*(2, 54) = 1.43, *p* = 0.247, *η*^2^ = 0.05) nor the time × electrode position interaction (*F*(2, 54) = 0.138, *p* = 0.805, *η*^2^ = 0.01) reached significance.

P3: Under the congruent condition, time also showed a significant main effect (*F*(1, 27) = 4.88, *p* = 0.036, *η*^2^ = 0.15). However, electrode position (*F*(2, 26) = 1.88, *p* = 0.172, *η*^2^ = 0.13) and the time × electrode position interaction (*F*(2, 26) = 0254, *p* = 0.778, *η*^2^ = 0.02) were not significant. In the incongruent condition, a significant main effect of time was observed (*F*(1, 27) = 5.23, *p* = 0.030, *η*^2^ = 0.16), with post hoc comparisons revealing that P3 amplitude decreased after TSD. A significant main effect of electrode position was also found (*F*(2, 26) = 3.83, *p* = 0.035, *η*^2^ = 0.23). Post hoc tests indicated that the amplitude at Pz was significantly higher than at P4 (*p* = 0.013), with no significant differences between P3 and Pz, or between P3 and P4. The time × electrode position interaction was not significant (*F*(2, 26) = 0.71, *p* = 0.502, *η*^2^ = 0.52).

**Figure 4:**
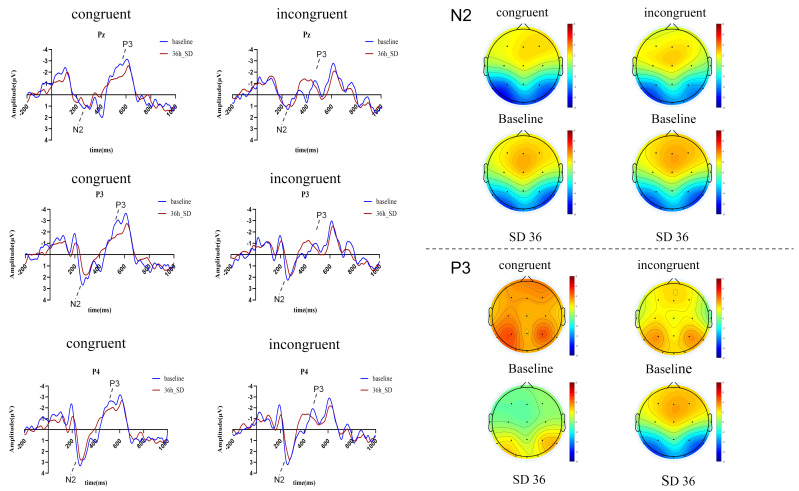
Waveforms and topographic maps of P3 and N2 at electrodes Pz, P3, and P4 under two conditions (before and after sleep deprivation). Phase difference analysis based on Hilbert transform showed no significant differences in parietal oscillatory phase between BL and TSD conditions during Incongruent processing (all *p* > 0.05), indicating that TSD modulates the amplitude (rather than the temporal phase) of parietal alpha/theta oscillations and N2/P3 components in working memory processing.

Peak values of N2 and P3 before and after sleep deprivation are presented in Table 4. Waveforms and topographic maps of the P3 and N2 components at electrodes Pz, P3, and P4 during the working memory task (before and after sleep deprivation) are shown in Figure 4.

## 4. Discussion

This study investigated the impact of acute total sleep deprivation (TSD) on working memory, focusing on changes in event-related electroencephalographic (EEG) activity in the parietal region (P3, Pz, and P4). Our findings reveal that working memory impairment following TSD is associated with weakened parietal alpha rhythm desynchronization (ERD) and deficits in both early conflict monitoring and late-stage attention evaluation processes.

Behaviorally, 36 h of acute TSD significantly impaired working memory ability, leading to decreased accuracy and prolonged reaction times. This impairment was consistent across both congruent and incongruent task conditions, underscoring the universal detrimental effect of acute sleep deprivation on working memory, in line with previous research [20].

In the alpha frequency band, we observed significantly enhanced parietal ERD after 36 h of sleep deprivation compared to baseline sleep (BL), under both congruent and incongruent working memory task conditions. This finding aligns with studies showing increased alpha-ERD during heightened cognitive load. However, the traditional interpretation of enhanced alpha-ERD as an indicator of active information processing [21] or effective resource mobilization [22] appears incongruent with our observed behavioral deficits. Emerging literature suggests that prolonged fatigue might lead to increased alpha power, reflecting diminished inhibitory function or attentional lapses [23]. Furthermore, studies have linked decreased alpha power with impaired task performance, possibly due to neural noise or loss of inhibitory control [24]. Consequently, the significant augmentation of parietal alpha-ERD under TSD likely does not signify a productive compensatory mechanism. Rather, it may reflect an over-taxed, albeit inefficient, mobilization of parietal resources in an attempt to maintain working memory function amidst fatigue. This could manifest as increased alertness, ultimately failing to compensate for impaired function and leading to task deficits. This suggests that enhanced parietal alpha-ERD under TSD may indicate an abnormal activation pattern, signifying disrupted inhibitory activity and a state of heightened alertness rather than efficient processing.

Furthermore, under both task conditions, theta-event-related synchronization (ERS) was significantly enhanced after 36 h of TSD. The theta rhythm is typically implicated in attention maintenance, memory encoding, and retrieval [25]. The observed increase in theta-ERS may reflect the brain’s attempt to recruit additional resources to sustain working memory performance. This aligns with findings where increased theta power correlated with heightened attention effort, as measured by pupillometry [26]. Consequently, this enhanced theta-ERS under sleep deprivation may represent increased effort rather than successful processing—an inefficient or inappropriate compensatory activation pattern indicating difficulty in maintaining cognitive function under fatigue.

At the event-related potential (ERP) level, we observed decreased amplitudes of the N2 and P3 waves after TSD in both congruent and incongruent conditions, supporting Hypothesis (3). This finding is consistent with studies on circadian rhythm adjustment [27]. The N2 wave is generally associated with advanced cognitive control processes, including error detection, task switching, and conflict resolution. A decrease in N2 amplitude suggests a weakening of early conflict monitoring efficiency and potentially impaired interference inhibition [28,29], requiring greater cognitive effort to process information. This implies that sleep deprivation compromises the brain’s early attention evaluation, reducing its efficacy in detection and processing critical information during working memory tasks.

The P3 wave is linked to evaluating stimulus significance, allocating attentional resources, and updating working memory [30]. In the incongruent condition, P3 amplitude exhibited a main effect of electrode position, with significantly lower amplitude at Pz compared to P4 (*p* = 0.013) post-TSD, and no significant differences between P3/Pz or P3/P4. The overall decrease in P3 amplitude after TSD indicates a reduction in the brain’s top-down attentional resource allocation. Individuals in a TSD state may struggle to effectively focus attention on task-relevant information, diminishing stimulus evaluation and updating efficiency, which directly impacts working memory operations. Collectively, the changes in N2 and P3 waves highlight the negative impact of sleep deprivation on attention. The N2 changes suggest reduced information processing efficiency in the early stages, while the P3 changes indicate diminished efficiency in attention resource allocation and utilization in later stages, both contributing to working memory impairment.

Our findings regarding the impact of sleep deprivation on N2 and P3 waves are consistent with numerous existing studies. Reductions in P3 amplitude are commonly interpreted as impaired executive functions, including attention and working memory deficits [10,31,32]. Peng et al. [33] specifically demonstrated prolonged N2 latency after 24 h TSD, correlated with prolonged reaction times in a two-back task, mirroring the trend observed here. Some research also suggests that sleep deprivation enhances N2 reactivity, reflecting increased cognitive resource mobilization for information processing [34], a compensatory mechanism also potentially at play in our study.

Moreover, the observed changes in ERP components (N2, P3) and parietal ERD/ERS demonstrated a degree of consistency. Specifically, after 36 h of TSD, enhanced parietal alpha-ERD and theta-ERS were accompanied by reduced N2 and P3 amplitudes. This synchronous change likely reflects a broader disruption in neural processing. The reduction in N2 amplitude is linked to diminished early conflict monitoring and interference inhibition [29]. The P3 component serves as a crucial index for cognitive resource allocation and task execution effectiveness [35]. Concurrently, theta oscillations are associated with task set selection and attention maintenance [36], and enhanced theta-ERS may represent a compensatory effort. A previously established link between P3 and alpha-ERD [37,38] further supports the notion that these ERP changes align with the cognitive processing difficulties reflected in neural rhythm adjustments—namely, the abnormal enhancement of alpha-ERD and theta-ERS. Together, these findings suggest adaptive adjustments in working memory-related neural rhythms post-sleep deprivation.

The impact of sleep deprivation on working memory is not attributable to a single neural mechanism but rather to comprehensive, coordinated changes between neural rhythm activities (ERD/ERS) and ERP components, collectively illustrating the interference of TSD with working memory-related neural processing.

In summary, this study deepens our understanding of how sleep deprivation affects brain function in the parietal region and its relationship with working memory impairment. It elucidates the roles of alpha and theta rhythms, as well as the N2 and P3 components, in TSD-induced working memory deficits, offering a novel perspective on the neural mechanisms underlying fatigue and sleep insufficiency’s impact on cognitive performance.

Despite the insights gained, several limitations of this study should be acknowledged. First, the sample size and the specific protocol for 36 h of acute TSD may constrain the generalizability of our findings to other sleep-loss paradigms, such as chronic sleep restriction or partial sleep deprivation. Future studies should explore whether these different forms of sleep loss yield distinct neuro-electrophysiological signatures. Second, while our findings highlight the role of parietal oscillations, the inherent spatial resolution of EEG limits the precise localization of brain activity. Integrating advanced neuroimaging techniques, such as fMRI or MEG, could provide a more comprehensive mapping of the functional interactions between alpha and theta rhythms across diverse cognitive domains. Furthermore, the present study focused on the immediate impact of TSD without including a recovery sleep condition. Investigating whether the observed behavioral and electrophysiological deficits are fully reversible after rest would significantly strengthen the interpretation of the identified biomarkers. Regarding neural dynamics, although we explored potential phase differences in oscillatory activity during incongruent processing, supplementary analyses revealed no significant phase shifts between baseline and TSD conditions. This lack of observed shift may be attributed to the acute nature of the 36 h paradigm and our strict control of circadian factors. Given that neural phase dynamics are intrinsically linked to biological rhythms, future research integrating circadian monitoring (e.g., melatonin measurements) and longer-term deprivation paradigms is warranted to clarify whether sleep-dependent modulation involves phase-specific neural timing adjustments. A further limitation is the restricted focus on parietal electrodes (P3, Pz, and P4) in the main analyses, without including frontal electrodes such as Fz and FCz. Although we recorded from a full 64-channel array, preliminary analyses revealed that neural oscillations at frontal sites showed less robust changes following total sleep deprivation (TSD) compared to the parietal region. To maintain a focused narrative on the key neuroelectrical indices underlying working memory impairment, we prioritized parietal data in this study. Future work should incorporate a whole-scalp electrode setup to further explore fronto-parietal network interactions in sleep-related cognitive deficits. Finally, while our results suggest that TSD-induced impairments involve compensatory activities—such as abnormal theta-ERS and alpha-ERD enhancements—the functional effectiveness of these mechanisms remains to be fully elucidated. Future research should determine whether enhanced alpha-ERD represents a loss of inhibitory control or an increase in neural noise. Additionally, quantifying the extent to which theta-ERS enhancement can successfully offset cognitive costs under fatigue will be crucial for developing targeted interventions.

## 5. Conclusions

This study elucidated the neuro-electrophysiological mechanisms underlying verbal working memory impairment induced by 36 h of total sleep deprivation (TSD) in healthy young adults by investigating specific parietal alpha-ERD, theta-ERS, and ERP components (N2/P3). The key findings confirmed that 36 h of TSD significantly impaired working memory performance—evidenced by decreased accuracy and prolonged reaction time—regardless of sex. These behavioral deficits were accompanied by abnormal enhancements of parietal alpha-ERD and theta-ERS at P3, Pz, and P4 electrodes, as well as reduced amplitudes and delayed latencies of N2 and P3 components. These results collectively indicate that TSD-induced working memory deficit is closely associated with disrupted parietal oscillatory dynamics, impaired early conflict monitoring, and decelerated attentional resource allocation processes.

This work extends previous research by identifying parietal alpha-ERD and theta-ERS as reliable neuroelectrical indices for evaluating TSD-related cognitive impairment. Specifically, the enhanced theta-ERS under TSD represents an inefficient neural compensatory mechanism under fatigue, while the latency shifts in ERPs suggest a slowdown in neural processing speed. The synchronous changes in parietal oscillatory activity and ERP components further enrich the understanding of the electrophysiological basis of sleep loss, providing novel evidence for the interaction between sleep regulation and working memory processing.

In conclusion, the present study clarifies the predominant role of parietal electrophysiological markers in TSD-induced working memory impairment. The identified indices not only provide a potential reference for evaluating cognitive deficits caused by sleep loss but may also serve as robust biomarkers for monitoring fatigue-related cognitive impairment in clinical and high-stakes occupational settings.

## Figures and Tables

**Table 1:** Means ± SD of behavioral indices in male (*n* = 14) and female (*n* = 14) participants.

	Condition	Sexual	BL	TSD	*F*	*p*	*η* ^2^
Accuracy (%)	Congruent	Male	0.870 ± 0.130	0.842 ± 0.114	1.809	0.157	0.095
		Female	0.929 ± 0.055	0.848 ± 0.126			
	Incongruent	Male	0.587 ± 0.178	0.540 ± 0.161	1.488	0.229	0.079
		Female	0.652 ± 0.071	0.589 ± 0.130			
RT (ms)	Congruent	Male	491.64 ± 149.79	598.63 ± 210.71	1.387	0.257	0.074
		Female	524.17 ± 108.13	592.54 ± 179.81			
	Incongruent	Male	548.65 ± 152.90	638.55 ± 176.82	1.371	0.262	0.073
		Female	595.50 ± 128.38	668.15 ± 199.34			

**Table 2:** Means ± SD of parietal ERD/ERS in male (*n* = 14) and female (*n* = 14) participants.

	Condition	Electrode	Male		Female		*F*	*p*	*η* ^2^
			BL	SD	BL	SD			
alpha-ERD	Congruent	P3	0.240 ± 0.343	−0.045 ± 0.710	0.214 ± 0.493	−0.243 ± 1.215	1.256	0.299	0.068
		Pz	0.202 ± 0.346	−0.132 ± 0.874	0.393 ± 0.618	−0.444 ± 1.544	2.098	0.112	0.108
		P4	0.319 ± 0.638	−0.126 ± 0.739	0.081 ± 0.508	−0.312 ± 1.171	1.598	0.201	0.084
	Incongruent	P3	0.311 ± 0.653	0.021 ± 0.637	0.165 ± 0.648	−0.323 ± 1.098	1.685	0.182	0.089
		Pz	0.228 ± 0.808	−0.052 ± 0.355	0.146 ± 0.266	−0.237 ± 0.506	1.772	0.164	0.093
		P4	0.281 ± 0.422	−0.094 ± 0.710	0.110 ± 0.603	−0.393 ± 1.270	1.772	0.164	0.093
theta-ERS	Congruent	P3	0.175 ± 0.463	−0.012 ± 0.444	0.220 ± 0.252	−0.259 ± 0.719	2.679	0.134	0.134
		Pz	0.176 ± 0.427	−0.044 ± 0.460	0.771 ± 1.995	−0.697 ± 1.215	3.519	0.021	0.169
		P4	0.169 ± 0.356	0.009 ± 0.387	0.206 ± 0.234	−0.554 ± 1.500	2.677	0.057	0.134
	Incongruent	P3	0.344 ± 1.352	−0.008 ± 0.375	0.163 ± 0.247	−0.310 ± 0.651	1.767	0.165	0.092
		Pz	0.228 ± 0.808	−0.052 ± 0.355	0.146 ± 0.266	−0.237 ± 0.506	2.201	0.099	0.113
		P4	0.098 ± 0.269	0.018 ± 0.304	0.185 ± 0.159	−0.144 ± 0.398	3.145	0.033	0.154

**Table 3:** Means and standard deviations of theta-ERS and alpha-ERD (%) in the working memory task under baseline (BL) and total sleep deprivation (TSD) conditions, with statistical significance of between-condition differences.

Condition	Index	Electrode	BL (*n* = 28)	TSD (*n* = 28)	*t*	*p*
Congruent	alpha-ERD	P3	0.227 ± 0.417	−0.144 ± 0.881	2.33	0.028 *
		Pz	0.298 ± 0.501	−0.209 ± 1.168	2.359	0.024 *
		P4	0.209 ± 0.571	−0.220 ± 1.250	2.309	0.029 *
	theta-ERS	P3	0.198 ± 0.366	−0.135 ± 0.599	2.547	0.017 *
		Pz	0.474 ± 0.484	−0.371 ± 0.961	2.387	0.024 *
		P4	0.187 ± 0.269	−0.273 ± 1.122	2.109	0.044 *
Incongruent	alpha-ERD	P3	0.238 ± 0.643	−0.151 ± 0.898	2.275	0.031 *
		Pz	0.434 ± 0.795	−0.372 ± 1.433	2.295	0.030 *
		P4	0.196 ± 0.518	−0.244 ± 1.021	2.306	0.029 *
	theta-ERS	P3	0.254 ± 0.958	−0.159 ± 0.544	2.066	0.049 *
		Pz	0.187 ± 0.591	−0.144 ± 0.439	2.416	0.023 *
		P4	0.142 ± 0.221	−0.063 ± 0.357	2.655	0.013 *

BL = baseline (pre-sleep); TSD = 36 h of total sleep deprivation (after-sleep); ERD = event-related desynchronization; ERS = event-related synchronization. *p* < 0.05 was considered statistically significant.* *p* < 0.05

**Table 4:** Means and standard deviations of N2 and P3 peak amplitudes (μV) in the working memory task under baseline (BL) and total sleep deprivation (TSD) conditions, with statistical significance of between-condition differences.

Index	Condition	Electrode	BL (*n* = 28)	TSD (*n* = 28)	*t*	*p*
N2	Congruent	P3	−1.921 ± 1.733	−1.209 ± 0.942	−2.241	0.033 *
		Pz	−2.032 ± 1.916	−1.125 ± 0.931	−2.292	0.030 *
		P4	−1.788 ± 1.569	−1.215 ± 0.889	−1.719	0.097
	Incongruent	P3	−2.454 ± 3.421	−1.255 ± 1.105	−2.447	0.021 *
		Pz	−2.305 ± 2.425	−1.071 ± 0.913	−3.055	0.005 **
		P4	−2.162 ± 2.496	−1.070 ± 0.985	−2.417	0.023 *
P3	Congruent	P3	3.231 ± 2.651	2.076 ± 1.089	2.058	0.049 *
		Pz	3.192 ± 2.190	1.972 ± 1.150	2.428	0.022 *
		P4	2.941 ± 2.414	1.894 ± 0.930	1.965	0.06
	Incongruent	P3	3.519 ± 3.590	1.988 ± 1.326	2.218	0.035 *
		Pz	3.090 ± 3.441	1.993 ± 1.063	2.576	0.016 *
		P4	3.130 ± 3.240	1.850 ± 1.045	1.988	0.057

BL = baseline (pre-sleep); TSD = 36 h of total sleep deprivation (after-sleep); ERP = event-related potential. *p* < 0.05, * *p* < 0.05, ** *p* < 0.01 were considered statistically significant.

## Data Availability

The data presented in this study are available on request from the corresponding author due to commitments made in the informed consent to protect participant privacy.
